# Guy’s Cancer Cohort: Guy’s Cancer Centre’s Real-World Evidence Programme 5 years later

**DOI:** 10.1016/j.esmorw.2025.100145

**Published:** 2025-05-13

**Authors:** C.L. Moss, B. Russell, G. George, J. Handford, E. Josephides, S. Green, A. Mera, K. Haire, A. Igra, M. Gladman, S. Hachlili, A. Davies, D. Smith, S. Dolly, P. Ross, M. Lei, E. Sawyer, C. Harrison, M. Kazmi, A. Rigg, M. Van Hemelrijck, Guy’s Cancer Real-World Evidence Steering Committee, F. Higgins, J. Timbres, J. Broad, D. Guest, U. Angkawinitwong, I. Barnett, C. Barretto, A. Williams, B. Challacombe, P. Cathcart, S. Hughes, D. Enting, R. Nair, D. Josephs, T. O’Brien, A. Bille, E. Karapanagiotou, J. Gossage, K. Owczarczyk, A. Qureshi, A. Lumsden, N. Maisey, S. Ngan, A. Schizas, R. Kristeleit, R. Simo, J.P. Jeannon, J. Hubbard, T. Guerrero-Urbano, D. Sarker, J. Geh, M. Lynch, M. Harries, R. Mallipeddi, S. Morris, A. Keyoumar, O. Al-Salihi, J. Brady

**Affiliations:** 1 National Institute of Health Research, Guy’s and St Thomas’ NHS Trust, London; 2 Breast Cancer Genetics, King’s College London, London, SE1 9RT; 3 Commercial Directorate, Guy’s and St Thomas’ NHS Trust, London; 4 Centre for Innovation, Transformation and Improvement, Guy’s and St Thomas’ NHS Trust, London; 5 Experimental Cancer Medicine Centre, King’s Health Partners, London; 6 Comprehensive Cancer Centre, Guy’s and St Thomas’ NHS Trust, London, UK; 1 King’s College London, School of Cancer and Pharmaceutical Sciences, Transforming cancer OUtcomes through Research (TOUR), London, UK; 2 Breast Cancer Genetics, King’s College London, London, UK; 3 South East London (SEL) Accountable Cancer Network, Guy’s and St Thomas’ NHS Trust, London, UK; 4 Centre for Innovation, Transformation and Improvement, Guy’s and St Thomas’ NHS Trust, London, UK; 5 The Artificial Intelligence Centre for Value Based Healthcare, Guy’s and St Thomas’ NHS Trust, London, UK; 6 Comprehensive Cancer Centre, Guy’s and St Thomas’ NHS Trust, London, UK

**Keywords:** cancer, real-world data, routine clinical data, research database

## Abstract

**Background:**

As the burden of cancer rises, the value of real-world data in addressing clinically relevant research questions is increasingly recognised. The Guy’s Cancer Real-World Evidence Programme, initiated in 2018 at Guy’s and St Thomas’ Trust (GSTT), informs decision making and local policy. Within this programme, the Guy’s Cancer Cohort (GCC) database has amplified real-world evidence (RWE) capabilities and improved data internalisation. GCC was renewed by the Research Ethics Committee in May 2023 (Ref: 23/NW/0105) and expanded to include patients investigated for new or recurrent cancer.

**Construction and content:**

GCC includes patients investigated, diagnosed, and/or treated for cancer, enabling case-control studies and the exploration of factors linked to cancer diagnosis. Annually, ∼8000 cancers are diagnosed at Guy’s Cancer Centre. Only pseudonymised data are processed, and the database aligns with the National Health Service national opt-out scheme. Clinical data captured include demographics, tumour characteristics, treatments, quality of life, and imaging data.

**Conclusions:**

GCC provides essential infrastructure to explore clinical, mechanistic, and supportive care research questions. Its renewal and expansion optimise data collection and linkage, improving patient care and outcomes. Applications for pseudonymised datasets can be directed to cancerdata@gstt.nhs.uk.

## Highlights

• GCC: RWE database with anonymised data on 8000+ annual cases.
• RWE impact: cohort data aided 40+ publications, shaping cancer care and policy.
• Data and infrastructure: cohort captures diverse data; EPIC will enhance integration.
• Access and collaboration: reviewed data requests enable research on clinical studies.

## Introduction

In the UK, cancer incidence continues to rise, with Cancer Research UK estimating that between 2016 and 2018, 375 400 new cases were diagnosed, equating to one every 2 min.^1^ Breast, lung, and prostate cancers accounted for over half of all cases, with more than a third occurring in people aged 75 years or older. Despite increasing incidence, mortality rates have decreased by 19% over the last decade, and survival rates have doubled over the past 40 years.^2^ Almost 3 million people currently live with cancer in the UK, a number predicted to reach 4 million by 2030.^3^

Real-world data, derived from routine care rather than controlled clinical trials, are increasingly pivotal in research and policy.^4^^,^^5^ The Food and Drug Administration defines real-world evidence (RWE) as ‘clinical evidence regarding the usage and potential benefits and risks of a medical product derived from analysis of real-world data’.^6^ More broadly, in the context of cancer research, RWE encompasses insights derived from diverse health care data sources including electronic health records (EHRs), patient registries, and wearable devices.^7^ These data capture patient demographics, treatments, outcomes, and disease progression in real-world settings, offering a comprehensive view of patient experiences outside clinical trial constraints. Unlike clinical trials, generating RWE requires assessing and integrating diverse data sources. It supports clinical research, guideline development, benchmarking, and quality improvement.

Guy’s Cancer Centre, a leading UK treatment and research facility, offers personalised cancer care and prioritises accurate data collection for patient safety and auditing. In 2018, Guy’s Cancer Cohort was established to enable research using RWE, aligning with the centre’s strategy.^8^ Its partnership with King’s College London has enriched datasets and accelerated research, especially during the coronavirus 2019 (COVID-19) pandemic. Data from the cohort supported >40 publications, including studies on cancer patient outcomes with COVID-19.^9^, ^10^, ^11^, ^12^, ^13^, ^14^, ^15^ Renewed ethical clearance in May 2023 (Ref: 23/NW/0105) expanded its scope to include patients investigated for cancer but diagnosed with benign conditions, facilitating case-control studies to investigate factors associated with cancer diagnosis.

Guy’s Cancer Cohort is a Health Research Authority-approved research database (REC reference: 23/NW/0105, IRAS project ID: 325735). The research database received favourable opinion from the North West—Haydock Research Ethics Committee on 16 May 2023.

## Construction and content

Patients included in Guy’s Cancer Cohort are those investigated, diagnosed, and/or treated for cancer at Guy’s and St Thomas’ National Health Service (NHS) Trust (GSTT). Any patient with a Guy’s and St Thomas’ specific hospital number who is investigated for, or diagnosed with, a new or recurrent cancer is eligible for inclusion in Guy’s Cancer Cohort. Patients under the age of 18 years are ineligible. Since Guy’s Hospital is a referral centre, Guy’s Cancer Cohort also includes patients from secondary and tertiary hospitals. On average, between 2018 and 2021, ∼8000 new cancers were diagnosed at Guy’s Cancer Centre per year. In 2021, 982 of these cases accounted for lung cancer diagnoses, 1006 breast diagnoses, and 1005 prostate cancer diagnoses. In 2022, 2156 patients were investigated for cancer at the vague symptom Rapid Diagnostic Clinic (RDC) and ∼10% of the cohort received a subsequent cancer diagnosis.

The NHS national data opt-out programme was implemented in April 2018 with support from the National Data Guardian, UK government and the Department of Health and Social Care. The national opt-out programme is based on a new consent, or opt-out model, which permits patients to opt out of their personal confidential data being used for purposes beyond their direct care. This national opt-out policy is considered and applied alongside existing data protection legislation and best practice to ensure that processing of personal data aligns with the principles of being fair, lawful, and transparent.

In the case of the Guy’s Cancer Cohort, only pseudonymised data are processed, and personally identifiable data are accessible only by named members of the direct care team and the Guy’s Cancer Cohort Steering Committee. Furthermore, only named members of the Guy’s Cancer Real-World Evidence Programme are able to reidentify any pseudonymised datasets which have been generated for research purposes. Guy’s Cancer Cohort aligns, however, with the NHS national opt-out scheme, as patients are provided with the opportunity to opt out of use of their anonymised data for purposes outside of their direct care via the local information governance department. GSTT patients who object to their information being utilised for any purpose outside of their direct care at this site are asked to write to the information governance department to register their objection. The trust utilises a web-based technical solution which enables the opt-out status of Guy’s and St Thomas’ patients to be checked, and any relevant data to be removed from the Guy’s Cancer Cohort research database before use in approved research studies.

Guy’s Cancer Cohort also includes any routinely collected pseudonymised clinical information obtained before the initiation date of the research database in April 2018. Any retrospective data included within Guy’s Cancer Cohort are checked using the web-based opt-out tool before analysis and removed from the database if the patient has opted out of use of their data. Once withdrawn, no routinely collected clinical information is utilised for any purposes outside of the direct care of patients.

### Data collation

**Figure 1:**
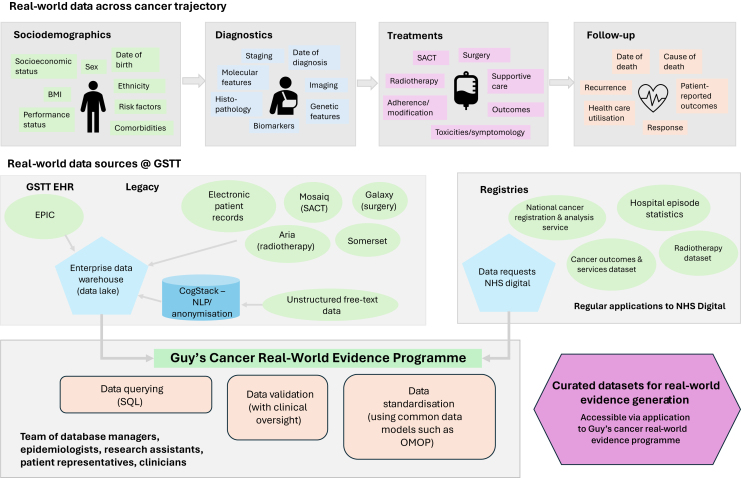
**Graphic to represent the real-world data points, data sources, and data-processing activities of the Guy’s Cancer Real-World Evidence Programme.** GSTT, Guy’s and St Thomas’ Trust; NHS, National Health Service; NLP, natural language processing; OMOP, Observational Medical Outcomes Partnership; SACT, systemic anticancer therapy.

Various clinical data are included in Guy’s Cancer Cohort including demographics, diagnostic assessments, tumour characteristics, treatment and intervention data, quality-of-life data [collected using patient-reported outcome measurements (PROMs)], and imaging data. A figure representing the various real-world data, data sources, and processing activities undertaken by the Guy’s Cancer Real-World Evidence Programme has been developed to provide further clarity (Figure 1).

Clinical data are captured from the various EHR systems of GSTT and incorporates both structured and unstructured information. CogStack, an application framework which permits the extraction of unstructured information from electronic clinical records, is utilised for these purposes across Guy’s Cancer Cohort.

Sociodemographic data include sex, date of birth, age at diagnosis, highest level of education, postal code (to estimate the deprivation index and carry out geographical analysis), ethnicity, body mass index (BMI), performance status, comorbidities, and any other relevant routinely collected pseudonymised data.

The following tumour characteristics are collected: TNM (tumour–node–metastasis) stage, grade, tumour diameter, number of tumours, histology and morphological codes and invasiveness, and any other clinical markers used to define tumour type and severity.

Treatment characteristics comprise data on the type and timing of treatment given (e.g. intravesical instillations, systemic chemotherapy, radical cystectomy, radiotherapy, or other treatments).

For surgical patients, the following pre-, peri-, and post-operative data are also collated:

**Table:** 

Preoperative	TNM stage, weight, height, BMI, American Society of Anesthesiologists (ASA) score, previous surgery, radiation, or neoadjuvant chemotherapy
Perioperative	Type of surgery, type of lymphadenectomy, blood loss, duration of surgery, accidental organ injury during surgery
Post-operative	Complications, re-operations, and re-admissions within 90 days, length of hospital stay, pT stage, number of excised lymph nodes, and number of excised and metastatic lymph nodes

Information on recurrence and survival is collected at regular intervals, using local patient identifiers, from the electronic patient records (EPRs).

Additionally, information on health-related quality of life is now collected for cancer patients using PROMs which are administered at regular intervals during treatment and follow-up.

At present, and as outlined within Figure 1, both legacy EHR and current real-world data from EPIC are fed into an enterprise data warehouse, which acts as a data lake within the GSTT environment. Within the Guy's Cancer Real-World Evidence Programme, we have database managers and analysts who work within this data lake environment using SQL coding to query various data sources and extract relevant clinical information. We then validate and transform these data with clinical input and, in some cases, apply a common data model or map to vocabularies to ensure a standardised format.

Currently, multiple different software systems (including EPR, patient information management system, and Somerset Cancer Register) are utilised concurrently to collate the routinely collected clinical information of Guy’s Cancer Cohort. However, GSTT are in the process of rolling out a centralised EHR platform which will ultimately replace all historical systems. All routinely collected clinical information will be reported from this EHR, EPIC, from October 2023. Based on the patients’ identifiers (hospital number, date of birth, surname, etc.), it is possible to link various systems. All identifiable information (i.e. hospital number, date of birth, and postcode), whether collected retrospectively or prospectively, is removed before releasing the data for research.

To enhance clinical interoperability and streamline data integration, we are working towards eventually incorporating EPIC data into our research database, in a more automated fashion. Our aspiration is to establish a seamless integration between EPIC and our research database repository, enabling bidirectional data exchange. This would facilitate the automatic transfer of relevant clinical information into the research database while ensuring compliance with data governance and security protocols. Automation of this integration would significantly reduce manual processes, improve data accuracy, and enhance the efficiency of RWE research. Future developments will focus on implementing application programming interfaces and data pipelines to facilitate direct interoperability between EPIC and research repositories, ultimately allowing real-time updates and reducing data latency.

We have also begun exploring the adoption of the Observational Medical Outcomes Partnership (OMOP) common data model and vocabulary to enhance the consistency and interoperability of our RWE datasets. Standardising our datasets using OMOP will improve integration with international research initiatives and enable larger-scale data analysis.

This initiative involves assessing the technical infrastructure needed for data transformation and mapping our existing formats to the OMOP vocabulary. Our goal is to ensure structured and semantically consistent data representation, making our datasets more accessible for clinical and epidemiological studies.

## Utility and discussion

**Figure 2:**
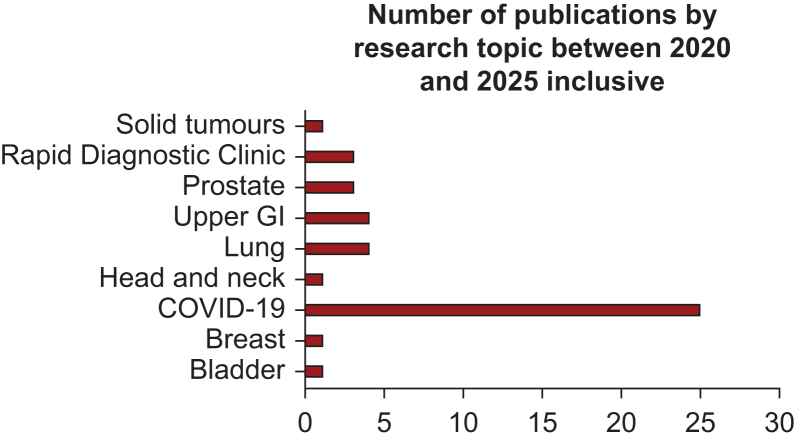
**Overview of the number of publications resulting from Guy’s Cancer Cohort per year, between 2020 and 2025.** COVID, coronavirus disease; GI, gastrointestinal.

**Figure 3:**
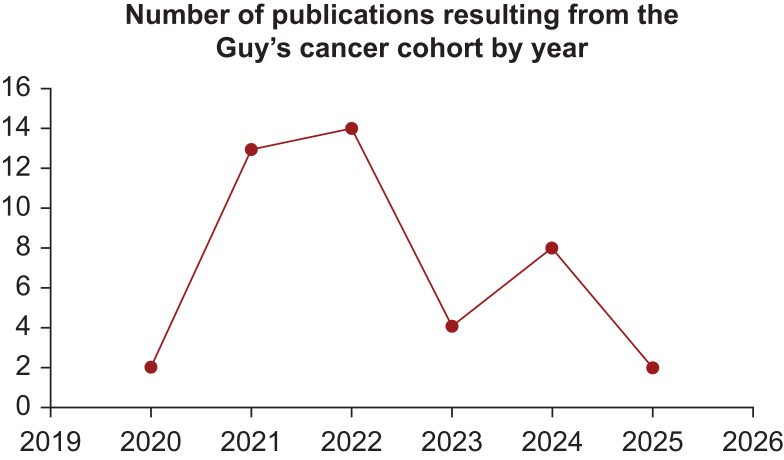
Graphic to describe the research topics published as part of Guy’s Cancer Cohort between 2020 and 2025.

To date, evidence generated as part of Guy’s Cancer Cohort has worked to improve the care, outcomes, and experience of cancer patients both at a local and national level. In 2022, there were 21 applications made to utilise the data of Guy’s Cancer Cohort including internally developed research projects, the contribution of data to national registries, and opportunities to commercialise the data. A variety of case studies are presented in the following sections to highlight the diversity of RWE research projects which can be facilitated by the Guy’s Cancer Cohort. Additionally, graphics presenting (i) the number of manuscripts published under the remit of the Guy’s Cancer Real-World Evidence Programme, and (ii) the number of publications by research topic between 2020 and 2025 are included in Figures 2 and 3, respectively, to provide further insight into the research output of this initiative. Moreover, a high-level overview of publication title, research topic, and year of publication is included within the Supplementary Material, available at https://doi.org/10.1016/j.esmorw.2025.100145.

### Radical cancer treatment safety during COVID-19 at Guy’s Cancer Centre^9^

This study evaluated the safety of radical cancer treatments during the first wave of COVID-19 at Guy’s Cancer Centre. Outcomes of patients treated during the pandemic were compared to pre-pandemic data. COVID-19 infection rates were low, with no related deaths. However, the number of patients receiving radical treatments dropped by 28% for surgery, 18% for systemic therapies, and 10% for radiotherapy. Despite these reductions, safety measures ensured a secure treatment environment for patients.

### Effectiveness of Guy's Rapid Diagnostic Clinic (RDC) in cancer detection^16^

Guy’s RDC, launched in 2016, investigates patients with non-specific symptoms unsuitable for site-specific referrals. Between 2016 and 2019, 1341 patients were assessed, with a cancer diagnosis rate of 7.2%. Median times to diagnosis and treatment initiation were 28 and 56 days, respectively. The study affirmed the RDC’s efficiency in diagnosing complex cases and highlighted the need for such streamlined pathways.

### Survival of young black patients with advanced non-small-cell lung cancer (NSCLC)

This Guy’s Cancer Centre study addressed the lack of data on NSCLC outcomes in black patients and young adults. Of 248 young NSCLC patients, 41 were black, with 37% being never smokers (defined as having smoked <100 cigarettes per lifetime). Median overall survival for black patients with metastatic disease was 12 months, similar to white patients, but far poorer than Asian patients (30.5 months). These findings underscore the need to engage black communities in clinical trials to improve representation and outcomes.

### Multidisciplinary team and access to data

The success of the Guy’s Cancer Real-World Evidence Programme is driven by a multidisciplinary team with expertise spanning data management, clinical research, governance, and patient advocacy. The core team consists of database managers and analysts who possess a detailed understanding of the data landscape, regulatory requirements, and the necessary approvals to access and process the data. Their expertise ensures that data are accurately curated, transformed, and maintained.

Epidemiologists also play a crucial role in facilitating real-world research by leveraging the curated data to support clinical teams in analysing outcomes, refining treatment guidelines, and ultimately improving patient care. They provide methodological expertise and statistical support, ensuring that research findings are robust and impactful.

A core group of tumour leads, or clinical champions, representing different tumour groups, provide further oversight of the research being conducted. Their clinical expertise is instrumental in validating datasets, ensuring that the extracted information is accurate, relevant, and reflective of real-world patient experiences. Their involvement bridges the gap between data science and clinical application.

The programme also benefits from the support of members from the Centre for Innovation, Transformation, and Improvement (CITI), who assist in managing data usage from both academic and commercial perspectives. They facilitate data-sharing agreements, ensure compliance with legal and ethical considerations, and oversee relevant contracts that enable collaborative research initiatives.

Finally, patient representatives are integral to the programme, ensuring that all research applications align with patient interests and ethical standards. Their involvement is critical in maintaining transparency and trust in data usage. Any proposed research must gain their approval before proceeding, reinforcing the programme’s commitment to patient-centred research and ethical integrity.

Access to Guy’s Cancer Cohort is coordinated by the project manager via cancerdata@gstt.nhs.uk. Detailed information, including an application form, is available on the GSTT CITI webpage. The Guy’s Cancer Real-World Evidence Programme welcomes detailed proposals from third parties.

### Application process

Initial appraisal is conducted by two epidemiologists from the Guy’s Cancer Cohort Steering Committee.

• Applications are reviewed by disease-specific access committees, each chaired by a tumour-lead clinician and including at least four clinical members. These committees assess the following:
  ○ Scientific merit, study design, funding details, and resource availability.
  ○ Strategic and collaborative value to King’s Health Partners (KHP).

The committees oversee the following:

1. Data requests (pilot/full studies).
2. Pilot study results (to approve continuation).
3. Study updates and extensions.

After initial review, applications are discussed and approved at monthly steering committee meetings, involving clinicians, patient representatives, database managers, and statisticians.

### Post-approval process

Once approved, the project manager liaises with contract teams, the commercial department, and information governance to finalise data transfer and sharing agreements before data release. Internal and external collaborators are eligible to apply for pseudonymised clinical data.

## Future perspectives

The Guy’s Cancer Real-World Evidence Programme is continually evolving, with several key initiatives in development to enhance our research capabilities. One major advancement is the establishment of a cardio-oncology database spanning Guy’s Cancer Centre and the Royal Brompton and Harefield Hospitals. This initiative aims to further elucidate cardiotoxicity experienced by cancer patients undergoing active treatment, facilitating improved monitoring and management strategies.

Additionally, we are leading the development and coordination of several national registries designed to capture real-world clinical data. These include a dihydropyrimidine dehydrogenase deficiency (DPYD) registry, which focuses on toxicity associated with fluoropyrimidine chemotherapy in patients with the mutant DPYD variant, as well as pipeline registries for rare cancers such as thymic cancer and mixed hepatocellular cholangiocellular carcinoma. These registries will enable contributing sites to submit data to our repository, fostering collaborative research and enhancing our understanding of treatment-related toxicities and outcomes.

Furthermore, we are actively engaged in various artificial intelligence (AI) initiatives aimed at leveraging imaging data to validate auto-contouring techniques and increase efficiency in radiotherapy planning. By integrating AI-driven tools, we aim to enhance the precision of radiation treatment, ultimately leading to improved patient outcomes and streamlined clinical workflows.

These future developments underscore our commitment to advancing RWE research, facilitating interdisciplinary collaboration, and leveraging technology to improve cancer care.

## Conclusions

The value of RWE continues to be recognised by regulatory agencies, academic institutions, and industry partners, and Guy’s Cancer Cohort provides the necessary infrastructure to address clinically relevant research questions of a mechanistic and supportive care nature. The renewal of the Guy’s Cancer Cohort research database ethics facilitates the continued optimisation of local data collection and linkage processes and impacts patient care, outcomes, and experience. Applications to support collaborative research are welcomed from all third parties.

## Appendix

**Guy’s Cancer Real-World Evidence Steering Committee**

F. Higgins^1^, J. Timbres^2^, J. Broad^3^, D. Guest^4^, U. Angkawinitwong^4^, I. Barnett^4^, C. Barretto^4^, A. Williams^5^, B. Challacombe^6^, P. Cathcart^6^, S. Hughes^6^, D. Enting^6^, R. Nair^6^, D. Josephs^6^, T. O’Brien^6^, A. Bille^6^, E. Karapanagiotou^6^, J. Gossage^6^, K. Owczarczyk^6^, A. Qureshi^6^, A. Lumsden^6^, N. Maisey^6^, S. Ngan^6^, A. Schizas^6^, R. Kristeleit^6^, R. Simo^6^, J. P. Jeannon^6^, J. Hubbard^6^, T. Guerrero-Urbano^6^, D. Sarker^6^, J. Geh^6^, M. Lynch^6^, M. Harries^6^, R. Mallipeddi^6^, S. Morris^6^, A. Keyoumar^6^, O. Al-Salihi^6^, J. Brady^6^

^1^National Institute of Health Research, Guy’s and St Thomas’ NHS Trust, London; ^2^Breast Cancer Genetics, King’s College London, London, SE1 9RT; ^3^Commercial Directorate, Guy’s and St Thomas’ NHS Trust, London; ^4^Centre for Innovation, Transformation and Improvement, Guy’s and St Thomas’ NHS Trust, London; ^5^Experimental Cancer Medicine Centre, King’s Health Partners, London; ^6^Comprehensive Cancer Centre, Guy’s and St Thomas’ NHS Trust, London, UK
