# Supplementary material for: Guy’s Cancer Cohort: Guy’s Cancer Centre’s Real-World Evidence Programme 5 years later
Source: ESMO Real World Data Digit Oncol. 2025 May 13;8:100145. doi: 10.1016/j.esmorw.2025.100145 (PMC12836791; doi:10.1016/j.esmorw.2025.100145)
Supplement: Supplementary data [file mmc1.docx]

Supplementary material:

*Overview of the output of the Guy’s Cancer Real-World Evidence Programme from 2020-2025 inclusive, including publication title, research topic investigated, and year of publication.*

| **Title** | **Research Topic** | **Year** |
| --- | --- | --- |
| A Retrospective Evaluation of PD-L1 Expression and Heterogeneity in Early-Stage Non-Small Cell Lung Cancer (REPLICA) | Lung | 2025 |
| Determinants of 5-year survival in patients with advanced NSCLC with PD-L1≥50% treated with first-line pembrolizumab outside of clinical trials: results from the Pembro-real 5Y global registry | Lung | 2025 |
| Treatment preferences of patients with muscle invasive bladder cancer: A discrete choice experiment | Bladder | 2024 |
| Anxiety and depression in patients with non-site-specific cancer symptoms: data from a rapid diagnostic clinic | Rapid Diagnostic Clinic | 2024 |
| The experience of surgical cancer patients during the COVID-19 pandemic at a large cancer centre in London | COVID-19 | 2024 |
| Real-world data evaluating Guy's rapid diagnostic clinic as an alternate pathway for patients with FIT levels below 10 | Rapid Diagnostic Clinic | 2024 |
| Predicting programmed death-ligand 1 (PD-L1) expression with fluorine-18 fluorodeoxyglucose ([18F]FDG) positron emission tomography/computed tomography (PET/CT) metabolic parameters in resectable non-small cell lung cancer | Lung | 2024 |
| Impact of SARS-CoV-2 vaccines and recent chemotherapy on COVID-19 morbidity and mortality in patients with soft tissue sarcoma: an analysis from the OnCovid registry | COVID-19 | 2024 |
| Previous immune checkpoint inhibitor therapy is associated with decreased COVID-19-related hospitalizations and complications in patients with cancer: Results of a propensity-matched analysis of the OnCovid registry | COVID-19 | 2024 |
| Prehabilitation exercise before oesophagectomy: long-term follow-up of patients declining/withdrawing from the program | Oesophageal | 2024 |
| The impact of 2 weeks wait referral on survival of head and neck cancer patients | Head & Neck | 2023 |
| Pathologic Lymph Node Regression After Neoadjuvant Chemotherapy Predicts Recurrence and Survival in Esophageal Adenocarcinoma: A Multicenter Study in the United Kingdom | Oesophageal | 2023 |
| SARS-CoV-2 omicron (B.1.1.529)-related COVID-19 sequelae in vaccinated and unvaccinated patients with cancer: results from the OnCovid registry | COVID-19 | 2023 |
| Safe delivery of systemic anti-cancer treatment for skin cancers during the COVID-19 pandemic | COVID-19 | 2023 |
| Non-Small Cell Lung Cancer (NSCLC) in Young Adults, Age < 50, Is Associated with Late Stage at Presentation and a Very Poor Prognosis in Patients That Do Not Have a Targeted Therapy Option: A Real-World Study | Lung | 2022 |
| Long-term effects of COVID-19 on cancer patients: the experience from Guy's Cancer Centre | COVID-19 | 2022 |
| The experience of UK patients with bladder cancer during the second wave of the COVID-19 pandemic | COVID-19 | 2022 |
| Radical cancer treatment is safe during COVID-19: the real-world experience of a large London-based Comprehensive Cancer Centre during the first wave | COVID-19 | 2022 |
| Impact of the COVID-19 pandemic on urological cancers: The surgical experience of two cancer hubs in London and Milan | COVID-19 | 2022 |
| Persistence of long-term COVID-19 sequelae in patients with cancer: An analysis from the OnCovid registry | COVID-19 | 2022 |
| Association between COVID-19 burden and delays to diagnosis and treatment of cancer patients in England | COVID-19 | 2022 |
| Safe provision of systemic anti-cancer treatment for urological cancer patients during COVID-19: a tertiary centre experience in the first wave of COVID-19 | COVID-19 | 2022 |
| COVID-19 Sequelae and the Host Proinflammatory Response: An Analysis From the OnCovid Registry | COVID-19 | 2022 |
| Mortality Among Adults With Cancer Undergoing Chemotherapy or Immunotherapy and Infected With COVID-19 | COVID-19 | 2022 |
| The Impact of COVID-19 on the Delivery of Systemic Anti-Cancer Treatment at Guy's Cancer Centre | COVID-19 | 2022 |
| The impact of hospital attendance on COVID-19 infection in cancer patients: an assessment of data from Guy's Cancer | COVID-19 | 2022 |
| Time-Dependent COVID-19 Mortality in Patients With Cancer: An Updated Analysis of the OnCovid Registry | COVID-19 | 2022 |
| Outcomes of head and neck cancer management from two cancer centres in Southern and Northern Europe during the first wave of COVID-19 | COVID-19 | 2022 |
| Comparison of Characteristics, Follow-up and Outcomes of Active Surveillance for Prostate Cancer According to Ethnicity in the GAP3 Global Consortium Database | Prostate | 2021 |
| COVID-19 in breast cancer patients: a subanalysis of the OnCovid registry | COVID-19 | 2021 |
| Prevalence and impact of COVID-19 sequelae on treatment and survival of patients with cancer who recovered from SARS-CoV-2 infection: evidence from the OnCovid retrospective, multicentre registry study | COVID-19 | 2021 |
| Predicting response to neoadjuvant chemotherapy in patients with oesophageal adenocarcinoma | Oesophageal | 2021 |
| Risk of COVID-19 death in cancer patients: an analysis from Guy's Cancer Centre and King's College Hospital in London | COVID-19 | 2021 |
| COVID-19 Vaccine Safety in Cancer Patients: A Single Centre Experience | COVID-19 | 2021 |
| Survival Outcomes in Invasive Lobular Carcinoma Compared to Oestrogen Receptor-Positive Invasive Ductal Carcinoma | Breast | 2021 |
| COVID-19 Risk Factors for Cancer Patients: A First Report with Comparator Data from COVID-19 Negative Cancer Patients | COVID-19 | 2021 |
| Guy's and St Thomas NHS Foundation active surveillance prostate cancer cohort: a characterisation of a prostate cancer active surveillance database | Prostate | 2021 |
| Continuity of Cancer Care: The Surgical Experience of Two Large Cancer Hubs in London and Milan | COVID-19 | 2021 |
| Adjuvant therapy following neoadjuvant chemotherapy and surgery for oesophageal adenocarcinoma in patients with clear resection margins | Oesophageal | 2021 |
| Presentation, follow-up, and outcomes among African/Afro-Caribbean men on active surveillance for prostate cancer: experiences of a high-volume UK centre | Prostate | 2021 |
| The effectiveness of the Guy's Rapid Diagnostic Clinic (RDC) in detecting cancer and serious conditions in vague symptom patients | Rapid Diagnostic Clinic | 2021 |
| Impact of age on the toxicity of immune checkpoint inhibition | Solid tumours | 2020 |
| Factors Affecting COVID-19 Outcomes in Cancer Patients: A First Report From Guy's Cancer Center in London | COVID-19 | 2020 |
